# Drugs Associated with Pediatric Cataracts: A Real-World Pharmacovigilance Study

**DOI:** 10.3390/children13020243

**Published:** 2026-02-09

**Authors:** Jiantong Du, Chen Xing, Zhiyue Zhang, Zonghui Ma

**Affiliations:** Department of Ophthalmology, Peking University First Hospital, Beijing 100034, China

**Keywords:** pediatric cataract, FAERS, disproportionality analysis, pharmacovigilance

## Abstract

**Highlights:**

**What are the main findings?**

- This study represents the largest and most up-to-date analysis of the FAERS database (spanning 20 years), identifying pharmacovigilance signals predominantly for glucocorticoids, immunosuppressants, monoclonal antibodies, cystic fibrosis transmembrane conductance regulator (CFTR) modulators, antineoplastic agents, an antiepileptic drug, and a colony-stimulating factor and strengthening their correlation with pediatric cataract formation.
- The data demonstrate that the administration of drugs is a critical determinant of pharmacovigilance signals, detecting exceptionally high disproportionate reporting for ophthalmic corticosteroids (difluprednate) and intravitreal chemotherapy that far exceeds systemic associations.

**What are the implications of the main findings?**

- The results underscore the need for enhanced ophthalmic vigilance for children receiving these agents, highlighting potential drug-specific risks that have previously been under-recognized.
- The identification of these specific signals invites further mechanistic research to elucidate the distinct pathophysiological pathways of drug-induced cataracts in the pediatric eye.

**Abstract:**

**Background**: Pediatric cataracts are a main cause of irreversible vision loss and a significant public health challenge. This study aimed to identify pharmacovigilance signals by analyzing large-scale data from the U.S. Food and Drug Administration Adverse Event Reporting System (FAERS). **Methods**: Using real-world data from FAERS (Q1 2004 to Q3 2025), we investigated associations between medications and pediatric cataracts. Following data standardization, signal detection was performed using multiple disproportionality analyses, including the Reporting Odds Ratio (ROR), Proportional Reporting Ratio (PRR), Bayesian Confidence Propagation Neural Network (BCPNN), and Multi-item Gamma Poisson Shrinker (MGPS). The time to onset was also evaluated. **Results**: Among 690,374 reports for individuals aged 0–17 years, 671 reports involving 232 drugs were reported with cataracts. Disproportionality analysis identified 24 drugs with significant signals, predominantly glucocorticoids (11/24), followed by immunosuppressants, monoclonal antibodies, cystic fibrosis transmembrane conductance regulator (CFTR) modulators, antineoplastic agents, an antiepileptic drug, and a colony-stimulating factor. Difluprednate showed the highest pharmacovigilance signal (ROR: 963.67; 95% CI: 316.27–2936.31; *n* = 4). Notably, CFTR modulators exhibited striking signals: ivacaftor (ROR: 30.75; 95% CI: 18.06–52.37; *n* = 14), elexacaftor-ivacaftor-tezacaftor (ROR: 15.58; 95% CI: 9.86–24.63; *n* = 19), and ivacaftor-lumacaftor (ROR: 13.2; 95% CI: 7.9–22.07; *n* = 15). **Conclusions**: This study provides a comprehensive large-scale pharmacovigilance profile of drug-induced pediatric cataracts, identifying agents with high-risk pharmacovigilance signals and underscoring the need for proactive ocular monitoring. These findings can inform clinical decision making and prevention strategies and guide future mechanistic research.

## 1. Introduction

Unlike the quiescent epithelium of the adult lens, the pediatric lens is a metabolically active, rapidly proliferating tissue that drives emmetropization and visual pathway maturation [1,2]. This biological distinction creates a unique vulnerability: while the adult lens may tolerate minor insults, similar disruptions in a child—specifically during the critical period of visual development—can cause deprivation amblyopia and permanent visual impairment, even if the optical axis is surgically restored [3,4]. Consequently, pediatric cataracts are not merely a “miniature” version of the adult disease but a distinct clinical entity with profound public health implications. Although less prevalent than age-related cataracts, the socioeconomic burden of childhood blindness is disproportionately high. Measured in “blind years,” the impact is comparable to adult cataracts due to the long life expectancy of affected children, compounded by the need for complex management strategies involving general anesthesia, long-term visual rehabilitation, and monitoring for secondary complications like glaucoma [4,5,6,7].

The etiology of pediatric cataracts is multifactorial, spanning genetic mutations, metabolic errors, and intrauterine infections. However, drug-induced lenticular toxicity represents a critical, preventable, yet poorly characterized cause. Children have long been termed “therapeutic orphans”; their exclusion from pivotal clinical trials means that a vast array of systemic medications are prescribed off-label, without age-specific safety data [8,9]. This gap is dangerous because their distinct pharmacokinetic profiles, immature blood–ocular barriers, and highly mitotic lens cells may predispose them to toxicity mechanisms that do not exist in mature eyes [10,11,12]. The cataractogenic effects of corticosteroids have been well documented in the clinical literature; however, the ocular safety parameters of novel therapeutic agents—including monoclonal antibodies, targeted antineoplastic drugs, and psychotropic medications—remain poorly characterized within pediatric populations [12].

Current knowledge of drug-induced cataracts in children is primarily derived from the extrapolation of adult data [13], with a notable scarcity of post-marketing real-world data [14]. Addressing these gaps, this study performed a comprehensive real-world pharmacovigilance analysis of drug-induced cataracts specifically in the pediatric population (aged 0–17 years) using the FDA Adverse Event Reporting System (FAERS) database. We aimed to identify drugs with high-risk pharmacovigilance signals of pediatric cataracts among over 20,000 FDA-approved agents, providing ophthalmologists and pediatricians with evidence-based insights to balance therapeutic benefits against ocular risks and to implement timely surveillance strategies.

## 2. Materials and Methods

### 2.1. Study Design and Data Source

To perform this pharmacovigilance study, we conducted a retrospective, population-based disproportionality analysis utilizing real-world data from FAERS. FAERS is a publicly accessible database that aggregates spontaneous safety reports, including adverse events (AEs), medication errors, and product quality complaints, for FDA-approved drugs and therapeutic biologics. These reports are submitted voluntarily by healthcare professionals, consumers, and manufacturers worldwide. Given its extensive scale and rich informational diversity, FAERS serves as a key resource for post-marketing surveillance, enabling the detection of potential drug–AE associations in real-world clinical practice [15,16,17].

The FAERS data are publicly available and can be downloaded from the FDA website at https://www.fda.gov/ (accessed on 10 November 2025). We downloaded reports from Q1 of 2004 to Q3 of 2025 (Q1 refers to the first quarter, while Q3 refers to the third quarter) via the FAERS Quarterly Data Extract Files website; a total of 83 quarters were included in the analysis. There are seven different types of tables in the FAERS data files: DRUG, OUTC, RPSR, THER, INDI, REAC and DEMO. These tables, linked by a unique PRIMARYID (Unique number for identifying a FAERS report), provide comprehensive information on demographics, report sources, indications, drug exposure (drug name, route of administration), specific AEs and outcomes.

As the study utilized fully anonymized, publicly available data, requirements for ethical approval and informed consent were waived.

### 2.2. Data Standardization

AE terminology was standardized using the Medical Dictionary for Regulatory Activities (MedDRA), version 26.1. MedDRA, maintained by the International Council for Harmonisation (ICH), is globally adopted hierarchical terminology. Its structure organizes medical concepts across five levels, from broad to specific: System Organ Class (SOC), High-Level Group Term (HLGT), High-Level Term (HLT), Preferred Term (PT), and Lowest Level Term (LLT) [18]. For the purpose of AE identification and analysis, this study utilized the Preferred Term (PT) level, which represents the standard descriptor for specific medical concepts and is widely used to categorize events by affected organ system.

Drug name standardization was performed by two ophthalmologists. They assigned each entry a standardized generic name by consulting authoritative sources (e.g., FDA NDC Directory, clinical pharmacology references). Salts and esters were consolidated to their base active ingredient. Combination products were recorded as unique entities. Spelling variants, abbreviations, and brand names were manually harmonized. For consistency, the generic names of medications were utilized for all analyses in this study.

### 2.3. Search Strategy

To capture a comprehensive set of reported cases, we queried the database for all PTs related to cataracts, with the specific PT name (MedDRA code) listed as follows: “Atopic cataract (10069649)”, “Cataract (10007739)”, “Cataract cortical (10007748)”, “Cataract nuclear (10007759)”, “Toxic cataract (10044135)”, and “Lenticular opacities (10024214)”.

To enhance the specificity of the drug–event association analysis, only drugs documented as “Primary Suspect” in the reports were included. Concomitant, secondary suspect, or interacting drugs were excluded, thereby minimizing noise from unrelated medications and strengthening the relevance and accuracy of the subsequent disproportionality signals. When a report listed multiple Primary Suspect (PS) drugs, we selected the drug with the lowest numerical value in the DRUG_SEQ field (the drug sequence number in the FAERS database) in the research analysis.

We restricted the age range of the study population to under 18 years. Patient age data in the FAERS database were extracted from the “AGE” and “AGE_COD” fields in the demographic file. The raw age values are reported with non-uniform units, as specified in the AGE_COD field (e.g., “YR” for years, “MON” for months, “WK” for weeks, “DY” for days). To standardize all ages into a common unit (years), we performed the following conversions based on the AGE_CODE: ages coded as “MON” were divided by 12, those coded as “WK” were divided by 52, and those coded as “DY” were divided by 365. Age entries coded as “DEC” (decades) were converted by multiplying by 10. Cases with AGE_COD entries of “YR” were used directly. Following this standardization, we applied an inclusion criterion, selecting only those cases with a calculated age greater than 0 years and less than 18 years for subsequent pediatric-focused analysis.

### 2.4. Data Cleaning

Due to the spontaneous reporting nature of the database, which may contain duplicate entries or reports that have been withdrawn/deleted, this study applied a hierarchical deduplication protocol recommended by the FDA. Specifically, based on the PRIMARYID, CASEID, and FDA_DT fields from the DEMO table, all reports were first sorted by CASEID, FDA_DT, and PRIMARYID. For reports sharing the same CASEID, the entry with the latest FDA_DT value was retained. If multiple reports had identical CASEID and FDA_DT values, the one with the largest PRIMARYID was kept. As a result, for cases where the same patient was reported multiple times, only the most recent report was retained for analysis.

The temporal relationship between drug exposure and AE onset was assessed using the START_DT (therapy start date) and EVENT_DT (AE onset date) fields. Reports with chronologically inconsistent dates (i.e., EVENT_DT preceding or equal to START_DT), as well as entries with missing or implausible temporal data, were excluded during quality control to ensure data integrity.

### 2.5. Signal Mining

After data cleaning, disproportionality analysis was performed to identify potential drug–AE associations by statistically comparing observed reporting frequencies against expected background rates. Associations were assessed using 2 × 2 contingency tables and four complementary algorithms: the Reporting Odds Ratio (ROR), the Proportional Reporting Ratio (PRR), the Bayesian Confidence Propagation Neural Network (BCPNN), and the Empirical Bayesian Geometric Mean (EBGM) (Table 1) [19]. Each method offers distinct advantages: ROR mitigates small-sample bias, PRR provides high specificity, BCPNN effectively integrates multi-source evidence, and EBGM is robust for rare-event detection [20,21,22,23]. To ensure high-confidence signal detection, a conservative consensus approach was adopted, whereby a signal was considered positive only if flagged by all four algorithms. Stronger statistical associations are indicated by higher resultant signal values.

### 2.6. Time-to-Onset Analysis

Time to onset (TTO) was derived from the difference between EVENT_DT (the date of the adverse event occurrence) and START_DT (the date treatment began). To evaluate the temporal risk profile of pediatric drug-associated cataracts, a Weibull distribution model was employed. This model is characterized by two key parameters: the scale parameter and the shape parameter. The scale parameter reflects the timing of event onset, with larger values corresponding to a later occurrence of adverse events. The shape parameter describes the behavior of the hazard rate: a value below 1 suggests a declining hazard (early-failure pattern), a value equal to 1 implies a constant hazard, and a value above 1 indicates an increasing hazard (wear-out pattern).

### 2.7. Statistical Analysis

All statistical analyses were performed by R software (version 4.3.1). Descriptive statistics were used to summarize the demographic and clinical characteristics of the patient cohort. A two-sided *p*-value of less than 0.05 was considered statistically significant.

## 3. Results

**Figure 1:**
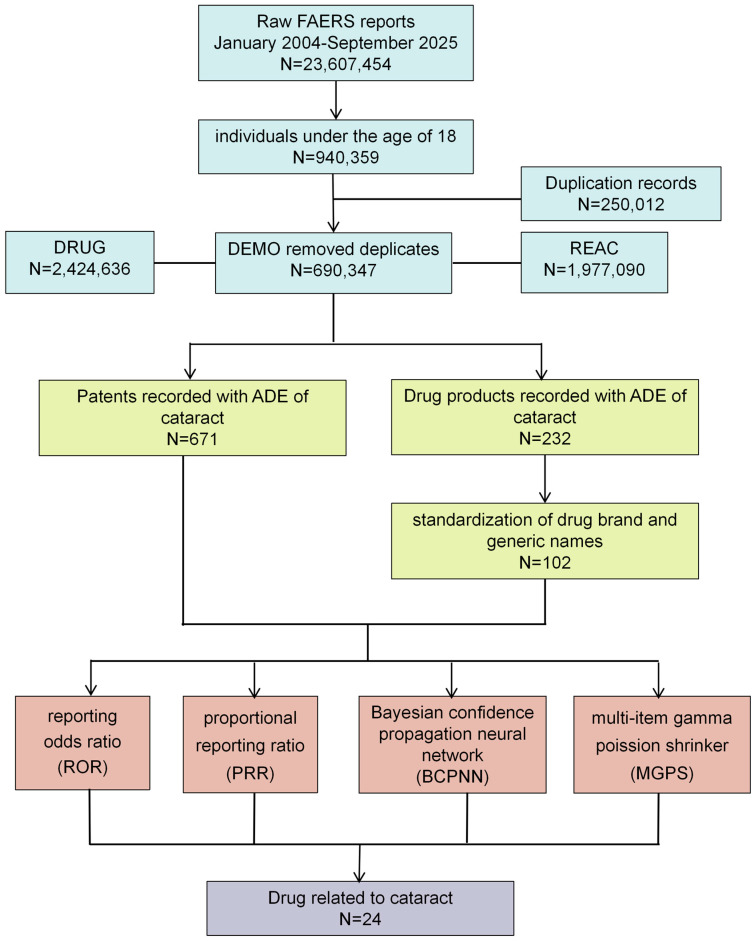
The flowchart of data mining.

**Figure 2:**
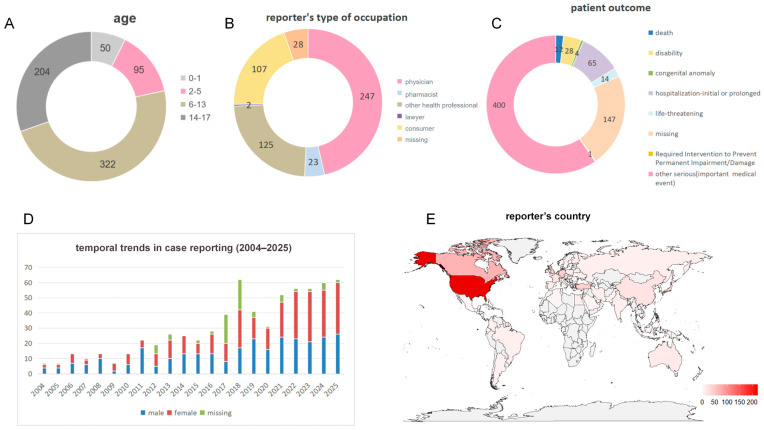
Characteristics of reports with cataracts. (**A**) Pie chart by age; (**B**) pie chart by reporter’s occupation; (**C**) pie chart by patient’s outcome; (**D**) histogram of the distribution of report year; (**E**) heatmap of the distribution by report country.

A total of 23,607,454 reports submitted from the first quarter of 2004 (2004Q1) to the third quarter of 2025 (2025Q3) were screened, of which 940,359 pertained to pediatric reports. After removing 250,012 duplicate reports, the remaining 690,347 reports constituted the final background population for the study. Among them, 671 (0.097%) were identified as cataract cases (Figure 1). More than half of the patients (n = 322) fell into the 6–13 year age group, followed by the 14–17 year group (n = 204) (Figure 2A). The overall outcome of the patients was generally poor, with 12 deaths reported and 400 patients experiencing outcomes classified as “other serious (important medical event)” (Figure 2C). Medical workers (including physicians, pharmacists and other health professionals) were the most frequent reporters of adverse events, accounting for 58.9% of all reports (Figure 2B). The year 2018 recorded the highest number of adverse event reports, totaling 62; no statistically significant difference was observed in the distribution of reports between males and females (Figure 2D). The United States submitted the highest number of reports (222 cases), followed by Canada (66 cases) and the United Kingdom (43 cases) (Figure 2E).

A total of 232 drugs were documented in adverse event records linked to pediatric drug-related cataracts. After standardization of generic and brand names, 102 unique drugs were included in a disproportionality analysis to evaluate their association with pediatric cataracts (Figure 1). The results of the disproportionality analysis for drugs with three or more reported cases are presented in Supplementary Table S1.

**Figure 3:**
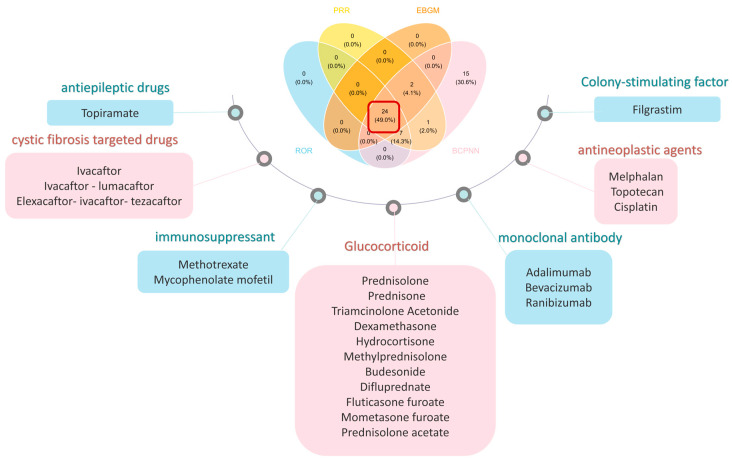
The identification and classification of drugs associated with pediatric cataracts. The red square in the picture represents the intersection of the four methods.

**Figure 4:**
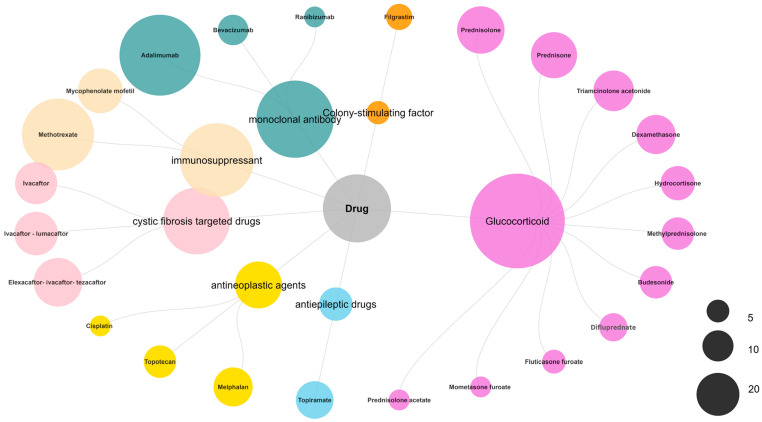
Grouped bubble chart of drugs associated with pediatric cataracts.

Ultimately, 24 drugs met the signal thresholds across all four algorithms (ROR, PRR, BCPNN, and EBGM) (Figure 3) and were subsequently categorized by their mechanism of action (Figure 4). Glucocorticoids accounted for a substantial proportion of the reports (99 cases), with prednisolone (18 reports), prednisone (17 reports), and triamcinolone acetonide (13 reports) being the most frequently reported. Monoclonal antibodies were also prominently represented, led by adalimumab (55 reports). Immunosuppressants, including methotrexate (43 reports) and mycophenolate mofetil (16 reports), were associated with cataracts. Additionally, cystic fibrosis-targeted therapies such as Elexacaftor/Ivacaftor/Tezacaftor (19 reports), Ivacaftor/Lumacaftor (15 reports), and Ivacaftor (14 reports) were identified. Antineoplastic agents melphalan (12 reports), topotecan (8 reports), and cisplatin (3 reports) also featured among the signals. The antiepileptic drug topiramate was associated with 11 reports.

The administration routes and indications for the above 24 drugs are detailed in Supplementary Table S2. Regarding the route of administration, the corticosteroids Difluprednate and Prednisolone acetate, as well as the antineoplastic agents Topotecan and Ranibizumab, are all administered via local ocular routes. The other drugs are primarily administered systemically. In terms of indications, Topotecan, Melphalan, and Ranibizumab are all indicated for ocular diseases. Specifically, both Topotecan and Melphalan are used for Retinoblastoma, while Ranibizumab is indicated for Retinopathy of Prematurity and Exudative retinopathies. The indications for the remaining drugs involve various systemic diseases.

**Figure 5:**
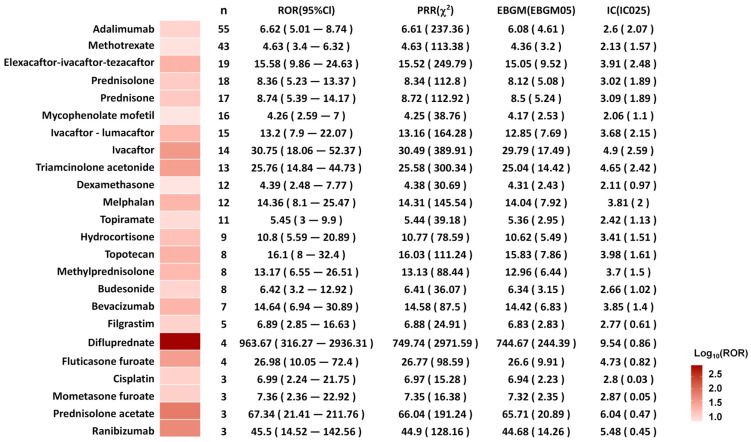
Heatmaps of drugs associated with pediatric cataracts. Abbreviations: ROR, Reporting Odds Ratio; PRR, Proportional Reporting Ratio; EBGM, Empirical Bayesian Geometric Mean; EBGM05, the lower limit of 95% CI of EBGM; IC, information component; IC025, the lower limit of 95% CI of the IC. Due to the extensive span of raw reporting odds ratios (RORs), data are visualized using the base-10 logarithm (logROR) to allow for clearer presentation of associations across different magnitudes.

The signals of drug–cataract associations were evaluated using the ROR value. Difluprednate showed the highest ROR for pediatric cataracts (ROR: 963.67; 95% confidence interval [CI] 316.27–2936.31; *n* = 4), followed by prednisolone acetate (ROR: 67.34; 95% CI 21.41–211.76; *n* = 3) and ranibizumab (ROR: 45.50; 95% CI 14.52–142.56; *n* = 3) (Figure 5).

**Figure 6:**
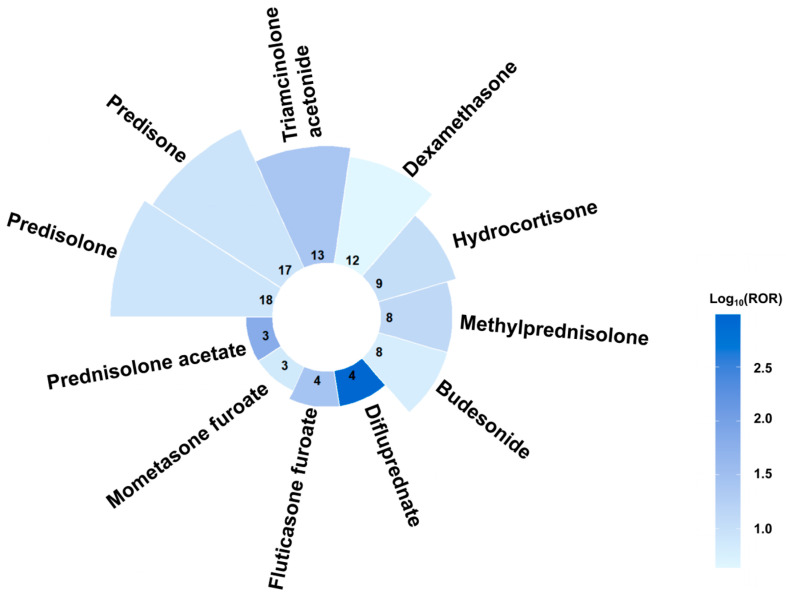
The integrated visualization of a bar chart and a heatmap for disproportionality analysis of glucocorticoids. Due to the extensive span of raw Reporting Odds Ratios (RORs), data are visualized using the base-10 logarithm (logROR) to allow for a clearer presentation of associations across different magnitudes.

Figure 6 integrates a bar chart and heatmap to visualize report counts and logarithmic ROR values across glucocorticoids. The top three drugs by report counts were predisolone, predisone, and triamcinolone acetonide, while the top three drugs by ROR values were difluprednate (ROR: 963.67; 95% CI 316.27–2936.31; *n* = 4), prednisolone acetate (ROR: 67.34; 95% CI 21.41–211.76; *n* = 3), and fluticasone furoate (ROR: 26.98; 95% CI 10.05–72.4; *n* = 4).

**Figure 7:**
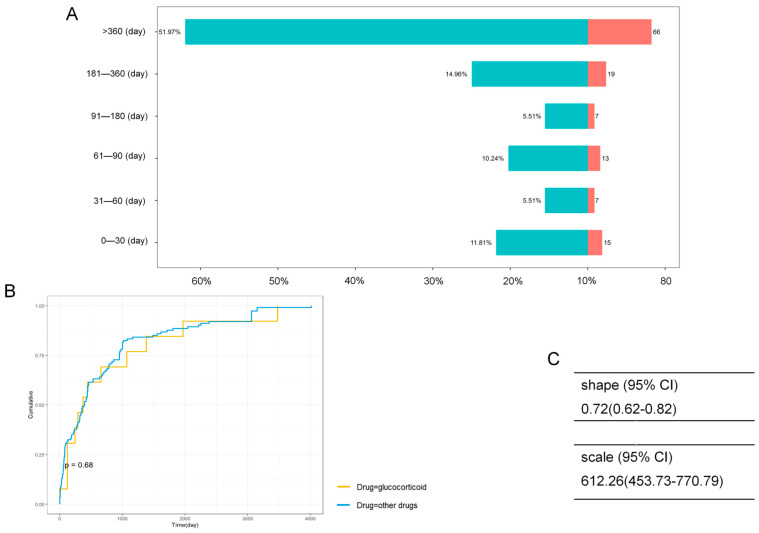
Time to event onset of drug-induced pediatric cataract. (**A**) Bi-direction bar chart of time to event onset; (**B**) Survival curve of time to event onset by drug group; (**C**) Weibull distribution of time to event onset.

After excluding reports with missing (482 reports) or incomplete (62 reports) data, a total of 127 (18.9%) reports were included in time-to-onset analysis. As shown in Figure 7A, the onset time varied widely, with the majority of cases occurring after 360 days from drug initiation (*n* = 66, 52.0%). No significant difference in onset time was observed between glucocorticoids and other drugs (Figure 7B). The Weibull distribution shape parameter was 0.72 (95% CI 0.62–0.82; Figure 7C). A shape parameter of less than one suggests that the risk of adverse events decreases over time, consistent with an early-failure distribution pattern.

We conducted a sensitivity analysis restricted to the narrow Preferred Term (PT) of “cataracts (10007739)” only, which yielded consistent results (Supplementary Table S3). CFTR modulators, ophthalmic corticosteroids and chemotherapeutic agents showed strong safety signals: Elexacaftor-ivacaftor-tezacaftor (ROR: 14.2; 95% CI 8.34–24.19; *n* = 14); Ivacaftor-lumacaftor (ROR: 13.11; 95% CI 7.38–23.27; *n* = 12); Ivacaftor (ROR: 24.36; 95% CI 12.57–47.2; *n* = 9); Difluprednate (ROR: 1199.03; 95% CI 393.23–3656.09; *n* = 4); Melphalan (ROR: 13.35; 95% CI 6.89–25.84; *n* = 9); Topotecan (ROR: 9.92; 95% CI 3.71–26.58; *n* = 4).

## 4. Discussion

While drug-induced cataracts are a well-documented adverse event (AE) in adults, the pediatric risk profile remains insufficiently characterized due to the exclusion of children from most clinical trials. Our retrospective analysis of the FAERS database (2004–2025) reveals a concerning upward trend in cataract reporting among pediatric patients, particularly in the post-2021 era. Unlike adults, the pediatric lens is in a critical stage of development; lens opacities during this period can obstruct the visual axis, leading to irreversible deprivation amblyopia if not managed promptly [24]. Our study bridges a critical knowledge gap by identifying novel signals, providing an evidence base for targeted ophthalmological surveillance.

Our analysis identifies a robust safety signal for CFTR modulators, a finding that significantly refines the current pharmacovigilance landscape. A study by Ali et al. (FAERS reports submitted between 2004 and 2024) identified a safety signal associating ivacaftor with pediatric cataracts [14], which aligns with the findings of our study. Compared to this study, our research incorporates more recent data (until 2025 Q3) and identifies high disproportionate reporting not just for ivacaftor (ROR: 30.75; 95% CI 18.06–52.37; n = 14) but crucially for the elexacaftor-ivacaftor-tezacaftor triple combination (ROR: 15.58; 95% CI 9.86–24.63; n = 19) and ivacaftor-lumacaftor (ROR: 13.2; 95% CI 7.9–22.07; n = 15). As the standard of care shifts toward these multi-drug regimens, characterizing their specific cataractogenic potential is essential.

These real-world findings provide critical translational validation of preclinical toxicology data, moving the evidence base beyond sporadic case reports [25]. Early safety studies by the manufacturer demonstrated that juvenile rats dosed with ivacaftor during the critical period of lens development (postnatal days 7–35) developed dose-dependent cataracts, a toxicity that was notably absent in adult rats [26]. Our findings suggest that this “window of susceptibility” is not species-specific but may translate directly to human infants. Mechanistically, this likely reflects the disruption of chloride and fluid homeostasis in the rapidly growing pediatric lens epithelium, caused by pharmacological modulation of CFTR channels.

Clinically, these results justify the strict implementation of the FDA’s recommendation for baseline and periodic ophthalmological examinations [27]. As CFTR modulators transform cystic fibrosis from a fatal pediatric condition into a manageable chronic disease, survival alone is no longer the sole metric of success; visual quality of life must be preserved. Unlike adults, young children face the irreversible risk of deprivation amblyopia if lens opacities are not detected and treated within the sensitive period of visual development. Consequently, the pharmacovigilance signal identified in our study argues that cataract surveillance should not be optional. We advocate for a standardized protocol—including baseline slit-lamp examinations prior to therapy initiation and annual follow-ups—to ensure that the survival benefits of these breakthrough therapies are not compromised by preventable visual disability.

Regarding antineoplastic agents, our analysis highlights how the route of administration determines ocular risk. We identified elevated safety signals for melphalan (ROR: 14.36; 95% CI: 8.1–25.47; *n* = 12) and topotecan (ROR: 16.1; 95% CI: 8–32.4; *n* = 8). These signals must be interpreted within the context of globe-salvaging retinoblastoma therapies (the indication for all these reports was retinoblastoma, as detailed in Supplementary Table S2), which prioritize intra-arterial and intravitreal delivery [28]. Unlike systemic chemotherapy, these routes expose the crystalline lens to high local concentrations of cytotoxic agents. This result is consistent with clinical research findings; intravitreal melphalan carries a documented dose-dependent cataract risk of 14–40% [29]. For cisplatin (ROR: 6.99; 95% CI: 2.2–21.75; *n* = 3), the signal likely reflects a multifactorial insult rather than a single etiology. In our dataset, cisplatin was primarily linked to medulloblastoma and nasopharyngeal carcinoma, malignancies necessitating craniospinal or head-and-neck irradiation—a potent cataractogenic factor [30]. However, rather than viewing cisplatin merely as a confounder, we hypothesize a synergistic toxicity. Cisplatin is a known oxidizing agent that depletes lenticular glutathione; this biochemical vulnerability likely lowers the threshold for radiation-induced oxidative stress, thereby accelerating cataractogenesis in this fragile population.

We identified a signal for filgrastim (granulocyte colony-stimulating factor, G-CSF). Current evidence does not support a direct cataractogenic mechanism for G-CSF. Instead, this likely represents confounding by co-medication. Filgrastim is prescribed to manage neutropenia in patients undergoing high-intensity chemotherapy regimens (e.g., conditioning for stem cell transplant) that include total body irradiation or high-dose alkylating agents [31]. Therefore, filgrastim serves as a clinical marker for a “high-risk therapeutic intensity” rather than a direct causative agent.

Corticosteroids are a known risk factor for inducing cataracts, which has been confirmed in previous FAERS studies primarily involving adult populations [13,32]. Notably, our results reveals that the route of administration influences the strength of the safety signal. We detected the ROR values for ophthalmic solutions—specifically difluprednate (ROR: 963.67; 95% CI 316.27–2936.31; *n* = 4) and prednisolone acetate (ROR: 67.34; 95% CI 21.41–211.76; *n* = 3)—which dwarf the signal for systemic prednisone (ROR: 8.74; 95% CI 5.39–14.17; *n* = 17). Known for its enhanced corneal penetration, difluprednate has been shown in comparative trials to induce posterior subcapsular cataracts more frequently than prednisolone acetate [33]. Therefore, the pharmacovigilance signals of topical ophthalmic corticosteroids warrant heightened clinical vigilance. We suggest implementing close ophthalmic follow-up for children requiring long-term use of topical corticosteroids.

We consider that signals for adalimumab, methotrexate, and mycophenolate mofetil arise from confounding by indication, not from direct drug influence. As cornerstones of therapy for JIA-associated uveitis [34,35], these agents are typically deployed in patients who already carry a high risk of cataracts and experience both chronic uncontrolled inflammation and the burden of topical corticosteroids. Consequently, these FAERS signals should not be misconstrued as evidence of causation; instead, they function effectively as epidemiological markers for severe, refractory uveitic disease.

The finding that over half (52.0%) of cases had an onset time exceeding one year is consistent with a mechanism of cataract involving slow, cumulative oxidative stress [36]. However, unlike adults, young children lack the verbal capacity to articulate subtle visual changes like glare or blur. Consequently, lens opacities are frequently identified only incidentally during routine wellness checks or, more tragically, after vision is severely compromised by leukocoria or strabismus. This inherent “detection lag” implies that the biological insult likely initiates significantly earlier than FAERS data suggests, reinforcing the necessity for proactive, scheduled slit-lamp screening rather than relying on symptom-based reporting.

The cumulative incidence curves for glucocorticoids and non-glucocorticoid agents (including CFTR modulators) were statistically indistinguishable (*p* = 0.68, Figure 7B). The temporal trajectory of risk appears remarkably conserved across pharmacological classes, suggesting that we can replicate the mature monitoring protocols for long-term glucocorticoid treatment [37] for patients receiving novel therapies like ivacaftor.

The shape parameter of the Weibull distribution of <1 (0.72) is characteristic of an “early failure” distribution [38], indicating a decreasing hazard over time. Our result showed a vulnerable subset of children (17.51%) develops opacities rapidly (0–30 days), suggesting that, for some patients, perhaps those with underlying metabolic defects or synergistic insults, the lens tolerance threshold is breached almost immediately.

Our study has limitations inherent to spontaneous reporting databases: underreporting and selective reporting bias may influence signal strength; FAERS data lack total usage denominators, preventing the calculation of true incidence rates; critical clinical details, such as cumulative dosage, exact duration of therapy, and lag time to onset, were frequently missing, precluding dose–response analysis; while disproportionality analysis detects statistical associations, it cannot establish causality. Furthermore, several limitations exist in the study design. First, to enhance accuracy, we only included reports where the drug-adverse event association was designated as “Primary Suspect (PS)” and defined a safety signal as one meeting the thresholds of all four disproportionality analysis methods concurrently. While this conservative approach improved specificity, it may have led to the under-reporting of some drug-associated pediatric cataract cases and other potentially related drugs. Given the overall low number of pediatric cataract reports, we considered the adoption of stringent criteria as justified; these findings could be validated in larger cohorts in the future. Second, the sample size available for Time-to-Onset (TTO) analysis was limited (n = 127). Considering the small size of analysis, as well as potential reporting delays and incomplete date fields in FAERS, the temporal association models derived therefrom require further confirmation with larger cohorts or more extensive datasets. Moreover, the TTO calculated from FAERS data reflects the interval between therapy initiation and event reporting rather than the true biological onset. This distinction is particularly crucial for pediatric cataracts, which are often asymptomatic in early stages and detected later via routine ophthalmologic screening. Therefore, the observed ‘early-failure’ pattern should be interpreted with caution, as it may be influenced by screening schedules and detection bias rather than biological susceptibility alone. Finally, due to the absence of data in the FAERS database regarding patients’ disease severity, history of drug exposure, and related clinical information, it is difficult to rule out the confounding effect of the disease severity itself on the results of this study. Therefore, the findings should be interpreted with caution and can only be regarded as pharmacovigilance signals with statistical significance. Further cohort studies or randomized controlled trials are still needed in the future to clarify the causal relationship between the drugs and adverse reactions.

## 5. Conclusions

This pharmacovigilance study delineates the landscape of drug-associated cataracts in children, confirming the pharmacovigilance signals of glucocorticoids (especially ophthalmic corticosteroids), immunosuppressants, monoclonal antibodies, cystic fibrosis transmembrane conductance regulator (CFTR) modulators, antineoplastic agents, an antiepileptic drug, and a colony-stimulating factor. For pediatric patients receiving CFTR modulators, ophthalmic corticosteroids, or localized chemotherapy for retinoblastoma, we advocate for the integration of rigorous, scheduled ophthalmological screening into clinical care pathways. Early detection is paramount to prevent amblyopia and ensure optimal visual rehabilitation in this developing population.

## Figures and Tables

**Table 1:** Principle of disproportionality measure and standard of signal detection.

Algorithms	Calculation Formula	Criteria
ROR	$ROR=\frac{a/c}{b/d}$ $95\%\;CI=e^{ln(ROR)\pm 1.96\sqrt{\frac{1}{a}+\frac{1}{b}+\frac{1}{c}+\frac{1}{d}}}$	a ≥ 3 and lower limit of 95% CI > 1
PRR	$PRR=\frac{a/(a+b)}{c/(c+d)}$ $95\%\;CI=e^{ln(PRR)\pm 1.96\sqrt{\frac{1}{a}-\frac{1}{a+b}+\frac{1}{c}-\frac{1}{c+d}}}$ $\chi^{2}=\frac{{\left(|ab-cd|-\frac{N}{2}\right)}^{2}\times N}{\left(a+b)(c+d)(a+c)(b+d\right)}$	a ≥ 3 and χ^2^ > 4
BCPNN	$IC=\log_{2}\frac{a(a+b+c+d)}{\left(a+b)(a+c\right)}$ $E(IC)=\log_{2}\frac{\left(a+\gamma 11)(a+b+c+d+\alpha )(a+b+c+d+\beta \right)}{\left(a+b+c+d+\gamma )(a+b+\alpha 1)(a+c+\beta 1\right)}$ $V(IC)=\frac{1}{{\left(ln2\right)}^{2}}\left\{\left[\frac{(a+b+c+d)-a+\gamma -\gamma 11}{\left(a+\gamma 11)(1+a+b+c+d+\gamma \right)}\right]+\;\left[\frac{(a+b+c+d)-(a+b)+\alpha -\alpha 1}{\left(a+b+\alpha 1)(1+a+b+c+d+\alpha \right)}\right]+\left[\frac{(a+b+c+d)-(a+c)+\beta -\beta 1}{\left(a+c+\beta 1)(1+a+b+c+d+\beta \right)}\right]\right\}$ $\gamma =\gamma \;11\frac{\left(a+b+c+d+\alpha )(a+b+c+d+\beta \right)}{\left(a+b+\alpha 1)(a+c+\beta 1\right)}$ $\mathrm{IC}-2\mathrm{SD}=E(\mathrm{IC})-2\sqrt{V(\mathrm{IC})}$ α1=β1=1;α=β=2; γ 11=1	E(IC) > 0
MGPS	$EBGM=\frac{a(a+b+c+d)}{\left(a+c)(a+b\right)}$ EBGM05=eln(EBGM)−1.95(1a+1b+1c+1d)^0.5	a > 0 and EBGM05 > 2

Abbreviations: a, number of reports containing both the target drug and cataracts; b, number of reports containing other adverse drug reactions of the target drug; c, number of reports containing cataracts and other drugs; d, number of reports containing other drugs and other adverse drug reactions. All data were cleaned prior to disproportionality analysis, with the deduplicated pediatric FAERS dataset forming the background population for signal detection. ROR, Reporting Odds Ratio; PRR, Proportional Reporting Ratio; BCPNN, Bayesian Confidence Propagation Neural Network; MGPS, Multi-item Gamma Poisson Shrinker (MGPS); 95% CI, 95% confidence interval; χ^2^, chi-squared; IC, information component; IC025, the lower limit of 95% CI of the IC; E (IC), the IC expectations; V(IC), the variance of IC; EBGM, empirical Bayesian geometric mean; EBGM05, the lower limit of 95% CI of EBGM.

## Data Availability

The data supporting the findings of this study were derived from the publicly available FDA Adverse Event Reporting System (FAERS) database (https://www.fda.gov/). All data extraction, cleaning, and analysis steps performed on the raw FAERS data are described in detail in the Methods section of this manuscript. The primary R code used in this study has been deposited in a public GitHub repository (https://github.com/dujiantong/FAERS.git).

## Supplementary Materials

The following supporting information can be downloaded at: https://www.mdpi.com/article/10.3390/children13020243/s1, Table S1: Disproportionality analysis for drugs with three or more reported cases; Table S2: Top Three Administration Routes and Indications; Table S3: Drugs associated with narrow PT definition (“cataract” only).

## Abbreviations

The following abbreviations are used in this manuscript:

AE	adverse event
BCPNN	Bayesian Confidence Propagation Neural Network
CFTR	cystic fibrosis transmembrane conductance regulator
FAERS	FDA Adverse Event Reporting System
G-CSF	granulocyte colony-stimulating factor
HLGT	High-Level Group Term
HLT	High-Level Term
ICH	International Council for Harmonisation
LLT	Lowest Level Term
MedDRA	Medical Dictionary for Regulatory Activities
MGPS	Multi-item Gamma Poisson Shrinker
PRR	Proportional Reporting Ratio
PT	Preferred Term
ROR	Reporting Odds Ratio
SOC	System Organ Class
TTO	Time to onset
