# Supplementary material for: Drugs Associated with Pediatric Cataracts: A Real-World Pharmacovigilance Study
Source: Children (Basel). 2026 Feb 9;13(2):243. doi: 10.3390/children13020243 (PMC12939617; doi:10.3390/children13020243)
Supplement: Supplementary file 1 [file children-13-00243-s001.zip › children-4113382-supplementary.pdf]

https://www.fda.gov/drugs/fdas-adverse-event-reporting-system-faers/fda-adverse-event-reporting-system-faers-latest-quarterly-data-files

Table S1. Disproportionality analysis for drugs with three or more reported cases.

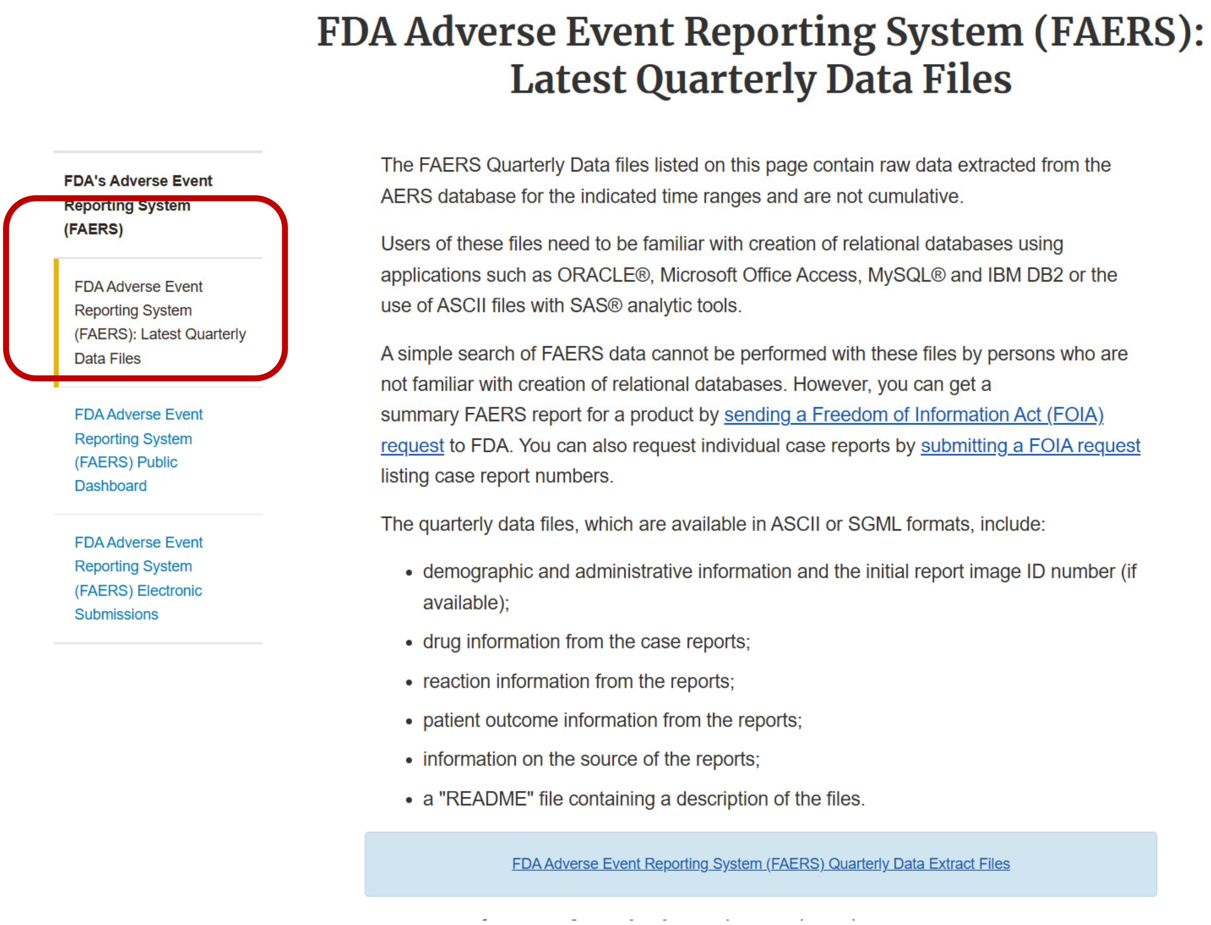

FDA Adverse Event Reporting System (FAERS): Latest Quarterly Data Files

FDA's Adverse Event Reporting System (FAERS)

- FDA Adverse Event Reporting System (FAERS): Latest Quarterly Data Files
- FDA Adverse Event Reporting System (FAERS) Public Dashboard
- FDA Adverse Event Reporting System (FAERS) Electronic Submissions

The FAERS Quarterly Data files listed on this page contain raw data extracted from the AERS database for the indicated time ranges and are not cumulative.

Users of these files need to be familiar with creation of relational databases using applications such as ORACLE®, Microsoft Office Access, MySQL® and IBM DB2 or the use of ASCII files with SAS® analytic tools.

A simple search of FAERS data cannot be performed with these files by persons who are not familiar with creation of relational databases. However, you can get a summary FAERS report for a product by sending a Freedom of Information Act (FOIA) request to FDA. You can also request individual case reports by submitting a FOIA request listing case report numbers.

The quarterly data files, which are available in ASCII or SGML formats, include:

- demographic and administrative information and the initial report image ID number (if available);
- drug information from the case reports;
- reaction information from the reports;
- patient outcome information from the reports;
- information on the source of the reports;
- a "README" file containing a description of the files.

FDA Adverse Event Reporting System (FAERS) Quarterly Data Extract Files

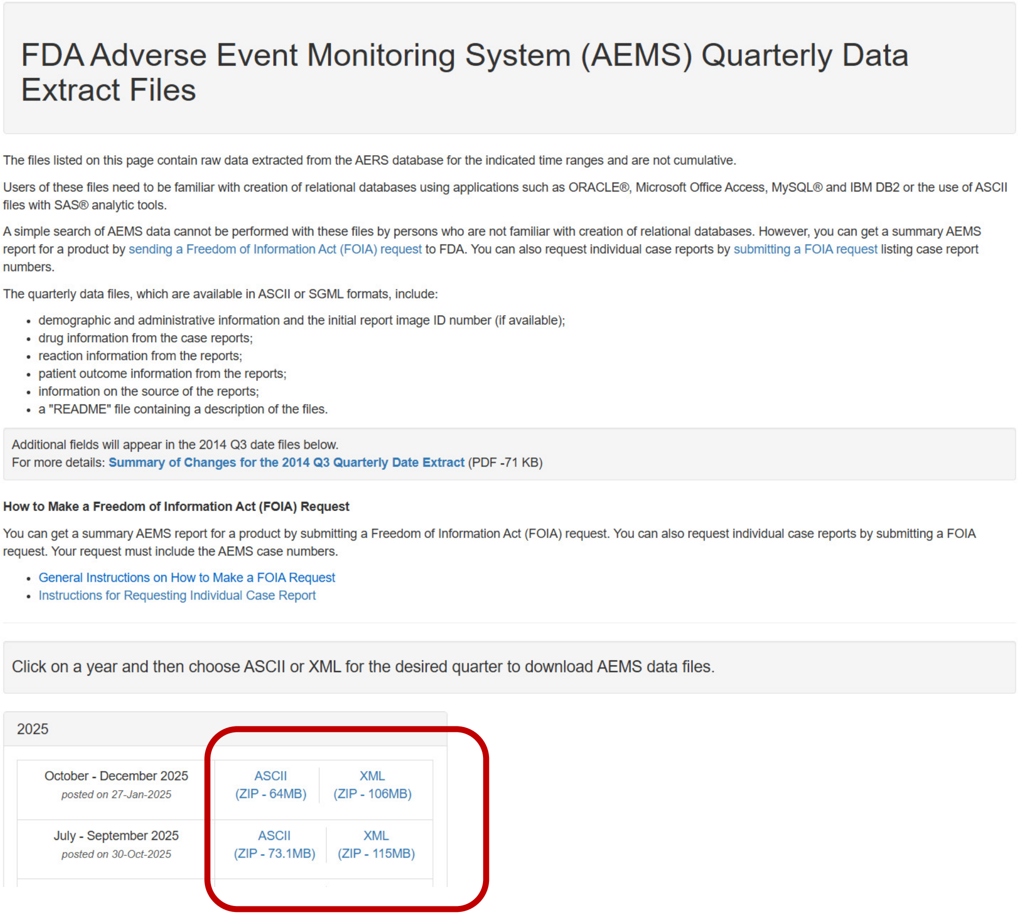

FDA Adverse Event Monitoring System (AEMS) Quarterly Data Extract Files

The files listed on this page contain raw data extracted from the AERS database for the indicated time ranges and are not cumulative.

Users of these files need to be familiar with creation of relational databases using applications such as ORACLE®, Microsoft Office Access, MySQL® and IBM DB2 or the use of ASCII files with SAS® analytic tools.

A simple search of AEMS data cannot be performed with these files by persons who are not familiar with creation of relational databases. However, you can get a summary AEMS report for a product by sending a Freedom of Information Act (FOIA) request to FDA. You can also request individual case reports by submitting a FOIA request listing case report numbers.

The quarterly data files, which are available in ASCII or SGML formats, include:

- demographic and administrative information and the initial report image ID number (if available);
- drug information from the case reports;
- reaction information from the reports;
- patient outcome information from the reports;
- information on the source of the reports;
- a "README" file containing a description of the files.

Additional fields will appear in the 2014 Q3 date files below.
For more details: **Summary of Changes for the 2014 Q3 Quarterly Date Extract** (PDF -71 KB)

How to Make a Freedom of Information Act (FOIA) Request

You can get a summary AEMS report for a product by submitting a Freedom of Information Act (FOIA) request. You can also request individual case reports by submitting a FOIA request. Your request must include the AEMS case numbers.

- General Instructions on How to Make a FOIA Request
- Instructions for Requesting Individual Case Report

Click on a year and then choose ASCII or XML for the desired quarter to download AEMS data files.

2025

October - December 2025<br>*posted on 27-Jan-2025*	ASCII<br>(ZIP - 64MB)	XML<br>(ZIP - 106MB)
July - September 2025<br>*posted on 30-Oct-2025*	ASCII<br>(ZIP - 73.1MB)	XML<br>(ZIP - 115MB)

Table S2: Top Three Administration Routes and Indications

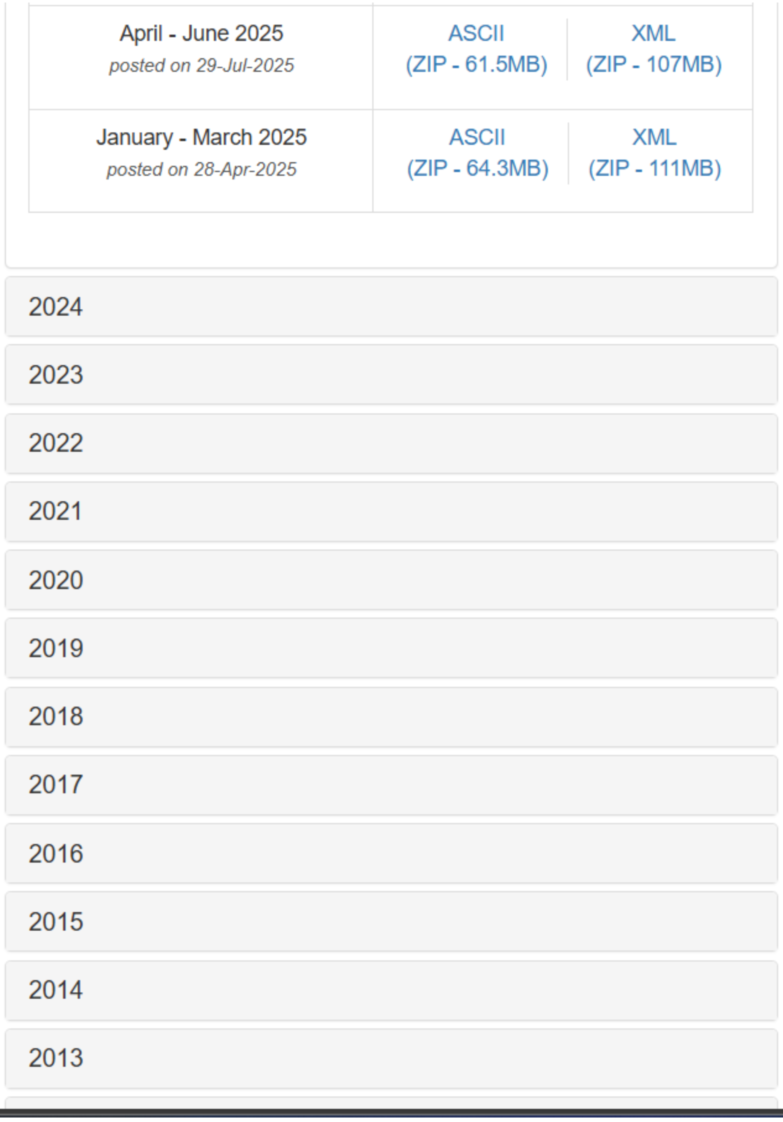

The quarterly data files based on your required time range can be downloaded.

Table S3: Drugs associated with narrow PT definition (“cataract” only)
